# Physiological and pathological functions of βB2-crystallins in multiple organs: a systematic review

**DOI:** 10.18632/aging.203147

**Published:** 2021-06-11

**Authors:** Meihui Li, Shengnan Liu, Wei Huang, Junjie Zhang

**Affiliations:** 1 Department of Obstetrics and Gynecology, Changhai Hospital, Naval Military Medical University, Yangpu, Shanghai 200433, China

**Keywords:** βB2-crystallin, physiological functions, pathological functions, tumor

## Abstract

Crystallins, the major constituent proteins of mammalian lenses, are significant not only for the maintenance of eye lens stability, transparency, and refraction, but also fulfill various physiopathological functions in extraocular tissues. βB2-crystallin, for example, is a multifunctional protein expressed in the human retina, brain, testis, ovary, and multiple tumors. Mutations in the βB2 crystallin gene or denaturation of βB2-crystallin protein are associated with cataracts, ocular pathologies, and psychiatric disorders. A prominent role for βB2-crystallins in axonal growth and regeneration, as well as in dendritic outgrowth, has been demonstrated after optic nerve injury. Studies in βB2-crystallin-null mice revealed morphological and functional abnormalities in testis and ovaries, indicating βB2-crystallin contributes to male and female fertility in mice. Interestingly, although pathogenic significance remains obscure, several studies identified a clear correlation between βB2 crystallin expression and the prognosis of patients with breast cancer, colorectal cancer, prostate cancer, renal cell carcinoma, and glioblastoma in the African American population. This review summarizes the physiological and pathological functions of βB2-crystallin in the eye and other organs and tissues and discusses findings related to the expression and potential role of βB2-crystallin in tumors.

## INTRODUCTION

Crystallins are ubiquitous, abundant proteins mainly found in the ocular lens, which is the tissue with the highest protein content in the human body [1, 2]. Their discovery dates to about 200 years ago, when Berzelius first identified and named the crystallins as specific entities of the bovine lens [3, 4]. In 1894, Morner successfully isolated three primary types, the α-, β-, and γ-crystallins, which proved to have highly heterogeneous patterns of expression in most vertebrate lenses [4–7]. These three classes were classified mainly by the sizes of the oligomers they form. The largest multimers, formed by α-crystallin, are on the order of 500 kDa. The β-crystallins represent a dimer- to octamer-sized mixture with molecular masses ranging from 45 to 180 kDa, while γ-crystallin monomers are approximately 20 kDa. The α-crystallins belong to the family of small heat-shock proteins (HSPs) which act as molecular chaperones during embryonic development [8–9]. The α-crystallin family comprises two subunits, referred to as αA- and αB-crystallins, which are encoded by the Cryaa and Cryab genes, respectively [10]. The classical function of α-crystallin is to serve as a chaperone, protecting the lens against stress conditions. However, studies showed that α-crystallins participate also in the protection and remodeling of the cytoskeleton, and contribute to inhibition of apoptosis through binding to pro-apoptotic Bcl-2 and Bcl-2 like 1 proteins [11–13]. The β- and γ-crystallins are thought to play a common structural role in the eye lens of vertebrates. These proteins share a common polypeptide chain fold, have conserved sequences, and are thus grouped into the βγ-crystallin superfamily, which is encoded by at least 14 genes [14, 15]. In mammals, these genes are not only organized as individual genes (Cryba1, Cryba2, Crygf, Crygs, CrygN), but also as duplets (Cryba4–Crybb1 and Crybb2–Crybb3) and into one major cluster (Cryga–Cryge) [11, 16]. The γ-crystallins are monomeric proteins with molecular masses of about 20 kDa, whereas the β-crystallins are a heterogeneous mixture of dimers and higher oligomers with native molecular masses ranging from about 50 kDa to 200 kDa [8]. Like all members of the βγ-crystallin superfamily, β-crystallins comprise two domains connected by an 8–10 amino acid inter-domain connecting peptide [17]. Each domain has two identical folded polypeptide chains, composed of a characteristic β-sandwich of two anti-parallel β-sheets conformations and N- and C-terminal extensions of varying lengths [17, 18]. Due to the similarity of these structures to paintings on ancient Greek pottery, they are known as ‘Greek key’ motifs [19, 20]. These motifs allow a dense packaging of the proteins to minimize light scattering, guaranteeing optimal transparency to the lens. For eye lens crystallins, the native folded state is required for lens transparency. In contrast, aggregated high molecular weight complexes are the source of light scattering leading to ocular pathologies. Seven β-crystallin genes, distributed on several chromosomes, code for homologous polypeptides termed βA1-, βA2-, βA3-, βA4-, βB1-, βB2-, and βB3-crystallin [7]. Four of the genes code for polypeptides with slightly lower isoelectric points, known as the βA (acidic) crystallins, while the other 3 encode the βB (basic) crystallins [4]. Unlike γ-crystallins, both acidic and basic β-crystallins have N-terminal extensions, whereas C-terminal extensions are found only in the basic polypeptides. Another difference between β- and γ-crystallins is that the two motifs comprising each β-crystallin domain are encoded by separate exons, whereas for γ-crystallins a single exon encodes both protein motifs [14].

## βB2-crystallin: more than a lens protein

One of the most prominent members of the vertebrate eye lens is the βB2-crystallin (gene symbol CRYBB2 in humans and Crybb2 in mice) [21]. The mouse Crybb2 gene is located on chromosome 5, within a cluster that includes three other Cryb genes. The corresponding human gene (CRYBB2) is mapped to chromosome 22q11.2 [22]. Crybb2 consists of six exons; the first one is untranslated, the second codes for the N-terminal extension, and the subsequent four exons code for the Greek key motifs [14, 23]. Although βB2-crystallin is expressed at negligible amounts in the embryonic mouse lens, its expression increases sharply at birth [24] to become the most abundant β-crystallin in the mouse lens by postnatal week 6 [25, 26]. Endogenous βB2-crystallin gene activity is upregulated in cultured lens cells by overexpression of β-catenin, which suggests a link between canonical Wnt-signaling and crystallin gene regulation [27]. Like α-crystallin, βB2-crystallin is also involved in cAMP-dependent and cAMP-independent phosphorylation pathways [28]. Studies demonstrated that the Greek key motifs of β-crystallins represent potential Ca^2+^-binding sites, which suggests a role for these proteins in Ca^2+^ buffering [29–31]. βB2 crystallin is the most energetically stable protein within the crystallin superfamily, capable of stabilizing and co-assembling other β-crystallins [32].

Until the 1990s, it was generally accepted that mammalian crystallins were evolutionarily highly conserved, lens-specific proteins. However, several discoveries changed this concept. Non-lenticular expression of α-crystallins is now well described, as the prominent expression of these proteins was demonstrated in rat spleen, thymus, rectum, cecum, liver, kidney, adrenal glands, cerebellum, and brainstem [13, 33]. In 1978, de Pomerai and Clayton provided one of the first reports on the presence of β-crystallin in non-lens tissue by demonstrating trace amounts of β-crystallin in a 60-day culture of 17-day embryonic chick neural retina [11]. In 1995, a more detailed study first reported clear evidence for the expression of βB2-crystallin in both murine and feline neural retina and retinal pigment epithelium (RPE), thus validating the presence in mammals of β-crystallin outside the lens [34]. These findings further contributed to challenging the original notion that crystallins are lens-specific proteins. Along with this evidence, it gradually took hold the concept that crystallins may originate in diverse cell types, pre-dating the evolution of the lens, and had a variety of functions before they were recruited to the lens to function as ‘crystallins’ [4]. Thus, research efforts eventually demonstrated that in addition to the ubiquitous crystallins found in the eye lens, some crystallins are essentially tissue-specific, while others have a completely separate machinery of expression in non-ocular tissues [35]. Indeed, stemming largely from studies in mice harboring mutations in Crybb2, such as the Philly [36], Aey2 [37], and 0377 [22] strains, we now know that the role of βB2-crystallin, the most important member of the β-crystallin superfamily, goes beyond its classical refractive function as a lens protein. Therefore, the purpose of this review is to illuminate the function of βB2-crystallin within and outside the lens.

## Physiological functions of βb2-crystallins

### Lens

Crystallins are highly soluble structural proteins that comprise 90% of the mammalian lens. Among them, βB2 crystallins are the most abundant β-crystallins in the human lens. The highly ordered, tightly packed crystallins make up the transparent structure of the lens and allow it to focus light onto the retina. To provide adequate lens structure and function, a protein must: (1) be highly soluble—high concentrations of soluble crystallins are responsible for the refractive index of the lens and maintain its transparency; (2) be extremely stable: the inherent stability of crystallins, arising from their native, compact structure, correlates tightly with their exceptional longevity; and (3) be able to have specific interactions with other crystallins: forming a stable protein matrix with a high degree of short-range order allows to increase resistance to oxidative stress and thermal denaturation, which is decisive to maintain lens transparency [8, 38, 39]. Therefore, the solubility and stability of βB2-crystallins are crucial determinants for the normal function of the lens: when these parameters are compromised, crystallin aggregation will affect lens transparency and reduce dioptric capacity [38–41]. Notable features of the molecular biology of the crystallin superfamily include the potential to be transported between cells via exosomes [42] and the ability of some of its members (e.g., αB- and βA3/A1-crystallins) to regulate lens differentiation and epithelial-mesenchymal transition (EMT) in RPE and tumor cells [43–45].

### Retina

A 2000 study demonstrated the expression of βB2-crystallin mRNA and protein in the mammalian retina [28, 46]. Subsequently, in 2007 a nonrefractive function of βB2-crystallin was first suggested by a study that indicated that this protein prevents the degeneration of the RPE and moves from the retinal ganglion cells (RGCs) into the extracellular space and retrogradely into the RGCs, although the underlying mechanisms remain to be elucidated [47]. Colocalization of βB2-crystallin with calmodulin, the major Ca^2+^-binding protein in the retinal ganglion cell (RGC) layer, provides further evidence that βB2-crystallins also operate via Ca^2+^ binding [48]. Immunohistochemical expression analyses in the retina, including filopodial protrusions and axons of adult RGCs, showed that βB2-crystallin is upregulated in the regenerating retina [49, 50] and promotes RGC survival after optic nerve axotomy through an autocrine mechanism [51]. In turn, cytoprotective functions of βB2-crystallin have been further demonstrated in cultured ARPE-19 (RPE) cells exposed to UV light, which showed increased viability and proliferation potential after addition of βB2-crystallin to the culture medium [47]. Indeed, evidence indicated that light-induced phosphorylation of β-crystallins mediates their anti-apoptotic chaperone activity in the RPE [52]. Of note, the latter function was further suggested in the uvea, as high expression of βB2-crystallins in retinal mitochondria was suggested to prevent cell death during the early stages of experimental autoimmune uveitis [53].

### Brain

Considerable evidence has accumulated over the past 20 years for the expression of various crystallins in several cell types and tissues, including the nervous system [54]. Gene analysis of a dominant cataract mouse model unmasked a crybb2 mutation and revealed that βB2-crystallin is expressed within distinct regions of the brain [22]. The Crybb2 transcript was best detected in the brain during postnatal development and through adolescence and was expressed predominantly in neurons of the olfactory bulb (mitral cell layer and glomerular layer), hippocampus (pyramidal cells of the CA1, CA2, and CA3 regions and granule cells of the dentate gyrus), cerebral cortex (pyramidal cells throughout all layers), and cerebellum (Purkinje cells and stellate cells of the molecular layers) [22, 55, 56]. As illustrated in animal models of optic nerve injury and axonal regeneration, mounting evidence highlights βB2-crystallin as a momentous factor that operates through autocrine and paracrine mechanisms to support axonal growth and repair, at least in part by accelerating the production of ciliary neurotrophic factor (CNTF) [49, 51, 57]. Furthermore, an important role for βB2-crystallins in synaptic remodeling was suggested based on evidence that these proteins facilitate dendritic outgrowth through regulating thymosin β4 (Tmsb4X) expression [58]. Thymosins play a crucial role in numerous cellular processes by affecting morphology, migration, and vesicle trafficking [59, 60]. All these properties emphasize the therapeutic potential of βB2-crystallins in the treatment of neurodegenerative diseases.

### Testis and ovary

Substantial evidence supports the expression of βB2-crystallin in both testis and ovary [61, 62]. Studies in the Philly mouse strain, which develops hereditary, progressive cataracts ~15 days after birth, led to identification of the crybb2^philly^ mutation as the responsible factor. The Crybb2^philly^ gene presents a 12-nucleotide in-frame deletion in the region encoding the fourth Greek key domain of the βB2-crystallin protein. Intriguingly, Philly mice were found to have poor fertility resulting from defective sperm and egg production [36, 62, 63]. Later on, the expression of βB2-crystallin was detected in spermatocytes from diverse mammals at the leptotene and zygotene stages [62, 64]. Indeed, βB2-crystallin transcripts are detected in the testis from birth throughout life and their expression is upregulated at postnatal day 17, consisting with the beginning of meiosis II [65]. Interestingly, a plausible connection between βB2-crystallin and infertility was provided by studies that identified βB2-crystallin as a microtubule-associated protein. This interaction can prevent microtubules from denaturation and impact sperm motility [66, 67]. After the generation of a βB2-crystallin null mouse (Crybb2^-/-^mouse) [68], further experiments allowed exploration of the mechanisms underlying subfertility caused by deficits in βB2-crystallin [69]. Evidence showed that decreased levels of Ca^2+^/calmodulin-dependent protein kinase IV (CaMKIV) in Crybb2^-/-^mice may affect the expression of Bcl-2, a major anti-apoptotic protein, which would reduce fertility by leading to abnormal proliferation and apoptosis of germ cells in the testis [70–72].

In the ovary, βB2-crystallin is mainly expressed in granulosa cells, with lower levels detected in theca cells [72]. It was reported that the progression of granulosa cells was inhibited in Crybb2^-/-^ mice, concomitant with decreased expression of two important cell cycle regulators, namely CDK4 and CCND2 [73]. In addition, in developing follicles the expression of Bcl-2 was distinctly lower after Crybb2 deletion, which demonstrated that βB2-crystallin influences female fertility by regulating granulosa cell apoptosis and follicular atresia [61, 74, 75]. Interestingly, further research indicated that downregulation of lncRNA A-30-P01019163 in ovary tissues from Crybb2^-/-^ mice may impair ovarian cell cycle and proliferation by reducing the expression of the purinergic receptor P2RX7 [76–78].

## Pathological functions of βb2-crystallin

### Ocular pathologies

In line with the main findings in the Crybb2 knockout mouse, several human studies attested to the association between mutations in the βB2-crystallin locus and cataracts [68, 79]. Besides a functional βB2-crystallin locus, in humans there is a second βB2-crystallin-derived pseudogene, termed CRYBB2P1. Conversion of the βB2 locus to the pseudogene results in lens opacification and cataract formation [80]. As shown in the Philly mouse model, misfolding of the mutated βB2-crystallin protein alters its aggregation properties, favoring the development of cataracts [81]. Subsequently, studies revealed additional amino acid-altering mutations in the CRYBB2 gene, in association with multiple types of congenital cataract, that result not only in structural changes in βB2-crystallin [82] but reduce also the solubility of these proteins to increase lens opacity [83–88]. Significant upregulation of βB2-crystallin occurs in several ocular pathologies, including age-related macular degeneration [89, 90], glaucomatous neuropathy [91], and cauterization-induced hypertension in rat model [91], and ocular hypertension in the rat [21, 90, 92].

### Neuropsychiatric disorders

Mutations in the mouse Crybb2 gene give rise to alterations in prepulse inhibition (PPI; an operational measurement of sensorimotor gating) and reduce hippocampal size, i.e., features typical of patients with schizophrenia [55, 93, 94]. Studies in mutant Crybb2^Philly^, Crybb2^Aey2^, and Crybb2^O377^ mice revealed C-terminal mutations of the βB2crystallin protein, likely associated with abnormal Ca^2+^ binding, which correlated with consistent alterations in adult behavior and evolution of neuropsychiatric disorders [56, 93, 95]. Notably, a meta-analysis of gene expression in the human cortex illustrated that the CRYBB2 gene shows the most significant association with five psychiatric disorders, namely attention-deficit hyperactivity disorder, autism, major depressive disorder, bipolar disorder, and schizophrenia [96, 97]. The distribution and function of βB2-crystallin in several organs are listed in Table 1.

**Table 1:** Distribution and function of βB2-crystallin.

**Organs**	**Expression**	**Regulation**	**Biological consequence**		**References**
			**Physiological**	**Pathological**	
lens	lens fiber cells	normal	maintain lenticular transparency and diopter		[8, 41, 42]
	mitochondria	up-regulated	anti-apoptosis		[44]
	lens fiber cells	mutant		multiple types of cataract	[68, 79] [87–89]
Retina	retinal pigment epithelium cells	normal	prevent degeneration		[47, 48]
	retinal ganglion cells	up-regulated	retina regeneration		[49, 50]
	retinal pigment epithelium cells	up-regulated	cytoprotective function		[47, 52]
	retinal ganglion cells	up-regulated		age-related macular degeneration	[90, 91]
	retinal ganglion cells	up-regulated		glaucomatous neuropathy	[92]
	outer plexiform layer of retinal ganglion cells	up-regulated		cauterization-induced hypertension	[92]
	retinal ganglion cells	up-regulated		ocular hypertension in rat	[21, 93]
Brain	retinal ganglion cells	up-regulated	axon regeneration		[51]
	ARPE-19 cells	up-regulated	epithelia-protection		[47]
	hippocampal neurons	up-regulated	axon formation		[57]
	hippocampal neurons	up-regulated	dendritic outgrowth		[57]
	hippocampal	mutant		Hippocampal abnormalities	[54, 94, 95]
	cortex	normal		attention-deficit hyperactivity disorder	[97, 98]
	cortex	normal		autism	[97, 98]
	cortex	normal		major depressive disorder autism	[97, 98]
	cortex	normal		bipolar disorder	[97, 98]
	cortex	normal		schizophrenia	[97, 98]
Testis	sperm	normal	maintain sperm motility		[61, 66]
	seminiferous tubule	normal	prevent microtubules from denaturation		[64, 67]
Ovary	granulosa cells and theca cells	gene knockout	subfertility		[69, 72]

## Cancer

### Breast tumors

In recent years, a potential role for CRYBB2 in carcinogenesis has been widely investigated. Research shows that African-American breast cancer patients have a higher risk of mortality than non-African-American patients [98, 99]. It has been proposed that the survival health disparity associated with breast cancer may be attributed to differences in tumor biology [100, 101]. As part of the Clinical Breast Care Project, Field et al. performed differential gene expression analysis in breast cancer samples and found that CRYBB2 had > 2.5-fold higher expression in African American compared to Caucasian women [102]. Of note, this finding was consistent with a previous study that combined CRYBB2 and PSPHL expression data to reliable distinguish African American from Caucasian breast cancer samples [103, 104]. Interestingly, a more recent study provided additional evidence that upregulation of the pseudogene CRYBB2P1, and not CRYBB2, is associated with race and poor outcome in breast cancer and possibly other tumors [102, 105]. Although molecular evidence is still inconclusive, these findings suggested that differential expression of CRYBB2/CRYBB2P1 contribute to poor outcomes in African American women by impacting tumor cell proliferation, invasion, metastasis, and tumor immunity [102].

### Colorectal cancer, prostate cancer, glioblastoma, and renal cell carcinoma

Similar to the breast cancer findings mentioned in the previous section, a 2008 study comparing gene expression profiles of prostate tumors from African American and Caucasian men pointed out a two-gene signature comprising CRYBB2 and PSPHL that accurately differentiated between these two groups [106]. Another report, dating back to 2012, described significant upregulation of CRYBB2 in colorectal cancer samples from African-American patients compared to European Americans [107]. In turn, a case-control association study reported that a genetic variant in the CRYBB2 gene (rs9608380) is associated with the risk of prostate cancer in African Americans [108]. Interestingly, a recent analysis identified CRYBB2 as one of 13 genes significantly associated with increased survival in African-American glioma patients in comparison to Caucasian ones [109]. Moreover, a study analyzing the significant disparities in survival between black and white patients with renal cell carcinoma showed that CRYBB2 was overexpressed in black patients associated in association with the WNT signaling pathway [110]. Altogether, these findings reaffirmed the notion that CRYBB2 expression in cancer is impacted by ethnicity [111]. Differences in βB2-crystallin expression between African American and non-African American cancer patients are summarized in Table 2.

**Table 2:** βB2-crystallin in African-American and non-African-American cancer patients.

**Types**	**Number**	**Regulated in African-American**	**Comparison items between African-American and Non-African-American**	**Outcome in African-American**	**References**
Breast cancer	52	up-regulated	age, size, grade, stage, ER status, subtype	poor	[108]
Breast cancer	161	up-regulated	age and stage	poor	[110]
Breast cancer	108	up-regulated	age, size, grade, ER status, subtype	poor	[109]
Colorectal Cancer	126	up-regulated	age, gender, location, stage	no mention	[112]
Prostate cancer	69	up-regulated	source, stage, gleason sum score, seminal vesicle invasionc, surgical margin status	no mention	[103]
Prostate cancer	527	up-regulated	age, PSA, family history	poor	[113]
Renal cell carcinoma	116	up-regulated	patients, performance score, smoking status, tumor laterality, clinical, pathologic	no mention	[115]
Glioblastoma	995	up-regulated	age, gender, KPS, histological type, G-CIMP status, person neoplasm cancer status, history of neoadjuvant treatment, targeted molecular therapy, radiation therapy, ethnicity	well (under the condition of KPS ≥ 80)	[114]

## Summary and prospect for CRYBB2 expression in tumors

High expression of CRYBB2/CRYBB2P1 is associated with higher breast cancer-related mortality in African-American women, likely in relation to enhanced tumor cell proliferation. Similarly, compared to Caucasians, upregulation of CRYBB2 is observed also in African-American patients with colorectal cancer, prostate cancer, renal cell carcinoma, and glioblastoma. Interestingly, dysregulated CRYBB2 expression is associated with poor outcomes in prostate cancer patients but correlates with better prognosis in African-American glioblastoma patients with Karnofsky performance score (KPS) ≥ 80. Still, for other tumor types and other populations, e.g., Asians, the correlation between CRYBB2/CRYBB2P1 expression and cancer progression and prognosis remains less certain. Although it remains unclear if and how a major lens protein would contribute to tumorigenesis, a likely connection may reside in the known regulation of less differentiation and crystallin expression exerted by the WNT signaling pathway [27, 112, 113], which is also a ubiquitous mediator of tumor growth and progression [114, 115]. Considering that no documented or hypothesized role for CRYBB2 in carcinogenesis has been explicitly put forward, it is conceivable that no causal relationship exists, at least for some malignancies, between high tumor CRYBB2 levels and tumor development. Clearly, mechanistic studies addressing potentially direct effects of β-crystallins on tumor cells are needed. Nevertheless, the documented association between CRYBB2 expression and multiple tumor types suggests that CRYBB2/CRYBB2P1 may serve as promising diagnostic or prognostic biomarkers in specific populations.

## CONCLUSIONS

βB2-crystallin, a main member of the βγ-crystallin superfamily, fulfills a key role in lens refraction and is also expressed in several extraocular tissues where it has distinct, non-lens functions. Besides functioning as a Ca^2+^-binding protein, βB2-crystallin is also involved in cAMP-dependent and cAMP-independent phosphorylation pathways. Notably, overexpression of either CRYBB2, the gene encoding for βB2-crystallin in humans, or its highly homologous pseudogene, CRYBB2P1, correlates with differential survival outcomes in African American patients with different malignant tumors. We hypothesize that βB2-crystallin contributes to poor diagnosis in malignancies such as breast and prostate cancer through regulating the TGF-β pathway or WNT signaling pathway and promoting epithelial to mesenchymal transition (EMT) [27, 116]. Considering the paucity of basic experimental research on the relationship between βB2-crystallin and tumorigenesis, a detailed exploration of the above mechanisms is needed to ascertain the role of βB2-crystallins in tumor development and metastasis.
